# Three-Dimensional Mass Spectrometry Imaging Identifies Lipid Markers of Medulloblastoma Metastasis

**DOI:** 10.1038/s41598-018-38257-0

**Published:** 2019-02-18

**Authors:** Martin R. L. Paine, Jingbo Liu, Danning Huang, Shane R. Ellis, Dennis Trede, Jan H. Kobarg, Ron M. A. Heeren, Facundo M. Fernández, Tobey J. MacDonald

**Affiliations:** 1 School of Chemistry and Biochemistry, Georgia Institute of Technology, Atlanta, GA 30332 USA; 2 Maastricht Multimodal Molecular Imaging Institute, Division of Imaging Mass Spectrometry, Maastricht University, Maastricht, 6229ER The Netherlands; 3 Aflac Cancer and Blood Disorders Center, Department of Pediatrics, Emory University School of Medicine, Atlanta, GA 30322 USA; 4 SCiLS Lab, Bremen, 28359 Germany; 5 Integrated Cancer Research Center, Georgia Institute of Technology, Atlanta, GA 30332 USA; 6 Institute of Bioengineering and Biosciences, Georgia Institute of Technology, Atlanta, GA 30332 USA

## Abstract

Treatment for medulloblastoma (MB) — the most common malignant pediatric brain tumor — includes prophylactic radiation administered to the entire brain and spine due to the high incidence of metastasis to the central nervous system. However, the majority of long-term survivors are left with permanent and debilitating neurocognitive impairments as a result of this therapy, while the remaining 30–40% of patients relapse with terminal metastatic disease. Development of more effective targeted therapies has been hindered by our lack of understanding of the underlying mechanisms regulating the metastatic process in this disease. To understand the mechanism by which MB metastasis occurs, three-dimensional matrix-assisted laser desorption/ionization mass spectrometry imaging (MALDI-MSI) experiments were performed on whole brains from a mouse model of human medulloblastoma. Analyzing the tumor and surrounding normal brain in its entirety enabled the detection of low abundance, spatially-heterogeneous lipids associated with tumor development. Boundaries of metastasizing and non-metastasizing primary tumors were readily defined, leading to the identification of lipids associated with medulloblastoma metastasis, including phosphatidic acids, phosphatidylethanolamines, phosphatidylserines, and phosphoinositides. These lipids provide a greater insight into the metastatic process and may ultimately lead to the discovery of biomarkers and novel targets for the diagnosis and treatment of metastasizing MB in humans.

## Introduction

Medulloblastoma (MB) is the most common malignant brain tumor of children, frequently disseminating throughout the central nervous system (CNS). Metastasis occurs in one third of patients at diagnosis and in two thirds at the time of relapse. The presence of metastasis is the most robust predictor of mortality among MB patients^1^. The need to reduce the risk of metastasis has driven the clinical development of aggressive treatment regimens that combine intensive chemotherapy with whole brain and spine irradiation that causes disabling neurotoxic side effects in the majority of long-term survivors^2^. The discovery of less toxic targeted therapies for the prevention of metastasis has been hindered by a lack of understanding of the mechanisms promoting dissemination and the molecular diversity of the disease. Identification of progression-associated metabolic phenotypes will lead to a better understanding of the pathophysiology of MB dissemination and can set the path for useful clinical outcomes such as biomarker diagnostic assays, more effective therapeutics, and personalized treatment regimens.

There are four distinct MB molecular subgroups, with the high-risk sonic hedgehog-activated subgroup (SHH MB) being of particular interest^3^. The presence of metastasis in this subgroup typically results in the poorest prognosis (~50% survival)^4^. To reliably investigate the mechanisms of metastasis in SHH MB, it is imperative to use *in vivo* models that faithfully recapitulate the initiation and progression of human SHH MB. These models must have as few genetic modifications as possible and be within the intact host immuno-microenvironment for the initiation and maintenance of metastasis^5^. The homozygous ND2:SmoA1 mouse, a transgenic model of human SHH MB derived from a constitutively active single point mutation of the mouse smoothened homolog expressed in mouse cerebellar granule neuron precursors is ideal for this purpose, with CNS metastases spontaneously occurring in 30% of mice, similar to humans^6^.

Several studies have sought to identify molecular drivers of metastasis in human or transgenic murine SHH MB. In our prior work, we identified mRNA overexpression of the Ras/MAPK pathway in association with human MB metastasis^7^. The Ras pathway was subsequently shown to confer treatment-resistance and promote metastasis in SHH-dependent tumors^8^. Jenkins *et al*. showed that ectopic expression of Arnt and Gdi2, which respectively play a critical role in fatty acid metabolism and Ras signalling, promoted tumor dissemination in SHH MB mice^9^. Recently, the Notch pathway transcription factor ATOH1 was overexpressed in murine SHH MB to demonstrate that dysregulated ATOH1 also promotes metastasis^10^. Interestingly, the Notch pathway, which regulates cell fate determination, was also found to play a key role in reprogramming mitochondrial metabolism^11^. The alteration to Ras pathway signalling, Arnt, Gdi2, and ATOH1 clearly promotes a different metabolic phenotype, however there has yet to be any reports identifying metabolomic changes associated with SHH MB metastasis.

In this investigation, we employ a label-free, high-throughput mass spectrometry imaging (MSI) approach to elucidate key lipid differences associated with primary tumors in the SmoA1 mouse model of SHH MB that have undergone metastasis relative to SmoA1 primary tumors that did not metastasize. The broad chemical detection afforded by MSI distinguishes it from other clinical imaging tools, such as MRI, PET, and SPECT that are limited to visualizing only one or a few specific molecules. Expansion of MSI experiments into three-dimensional space (3D-MSI) provides an even closer representation of the underlying biological structure and is particularly important for biological systems that display molecular heterogeneity, a hallmark of many cancer tissues.

There are several techniques able to perform 3D-MSI, of which matrix-assisted laser desorption/ionization (MALDI) is the most widely utilized. for biological samples. Latest generation MALDI-ToF-MSI instrumentation is now capable of much faster experiments, acquiring spectra at a rate of 50 pixels/s by combining high repetition-rate lasers synchronized with fast-moving sample stages while sustaining sufficiently-high data write speeds^12^. These MALDI-ToF-MSI instruments also provide the required spatial resolution (50 µm × 50 µm), mass resolution (~20,000 FWHM at *m/z* 800), and MS/MS capabilities needed to visualize changes in lipid distributions across different tissue regions within a mouse brain.

Understanding the underlying mechanisms of tumor progression in MB is key to enabling more accurate prognoses, personalized therapeutic regimens, and the development of novel therapeutics. Using 3D-MALDI-ToF-MSI, we identified unique lipids differentially expressed in a metastasizing tumor based on changes in their relative abundance compared with a non-metastasizing tumor. Such lipids provide a better understanding of MB tumor progression and, when validated, could lead to a panel of new diagnostic biomarkers and novel targets for the prevention of metastasis.

## Results and Discussion

**Figure 1:**
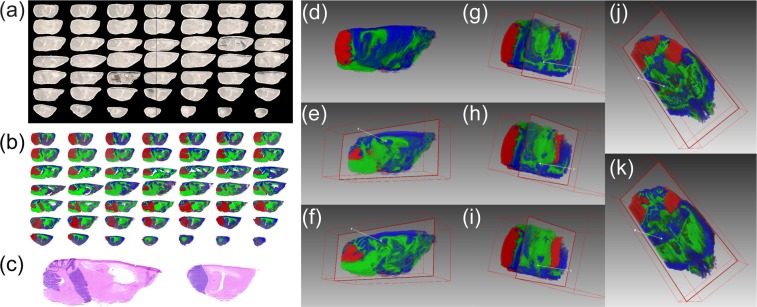
(**a**) Optical images of the 49 aligned sagittal sections from a ND2:SmoA1 transgenic mouse brain containing a non-metastasizing cerebellum tumor with (**b**) the corresponding MS visualization using three representative channels; *m/z* 790.5 in blue, *m/z* 888.6 in green, and *m/z* 885.5 in red, overlaid on one another. (**c**) Hematoxylin and eosin (H&E) stains of sections no. 29 and 41 indicating the primary MB tumor region (purple). (**d**) Three-dimensional alignment of all 49 mouse brain sections shown in (**b**). (**e–k**) Visualization of two sagittal (**e**,**f**), three coronal (**g–i**), and two transversal (**j**,**k**) virtual sections.

To investigate metastasis within SHH MB *in vivo*, whole brains from SmoA1 mice in which primary and metastatic tumor growths can be visualized by a green fluorescent protein tag (SmoA1-GFP) were imaged (Fig. 1**)**. These mice express a constitutively active form of Smoothened in cerebellar granule neuron precursors resulting in activation of the SHH pathway and MB tumor formation^13^. The disease progression in these mice faithfully recapitulates the pattern of tumor progression observed in humans with metastasis occurring in approximately 30% of animals^6^. After the mice were sacrificed, the brain and spinal cords were removed and inspected using fluorescence microscopy, allowing classification of primary tumors as being metastasizing or non-metastasizing (SI Fig. 1). Serial sections of the mouse brains were imaged using a Bruker rapifleX MALDI Tissuetyper ToF mass spectrometer capable of acquiring spectra at up to 50 pixels/s by the use of a 10 kHz laser and two galvanometric mirrors, allowing the laser beam to synchronously raster the sample while the sample stage rapidly moves^12^.

**Figure 2:**
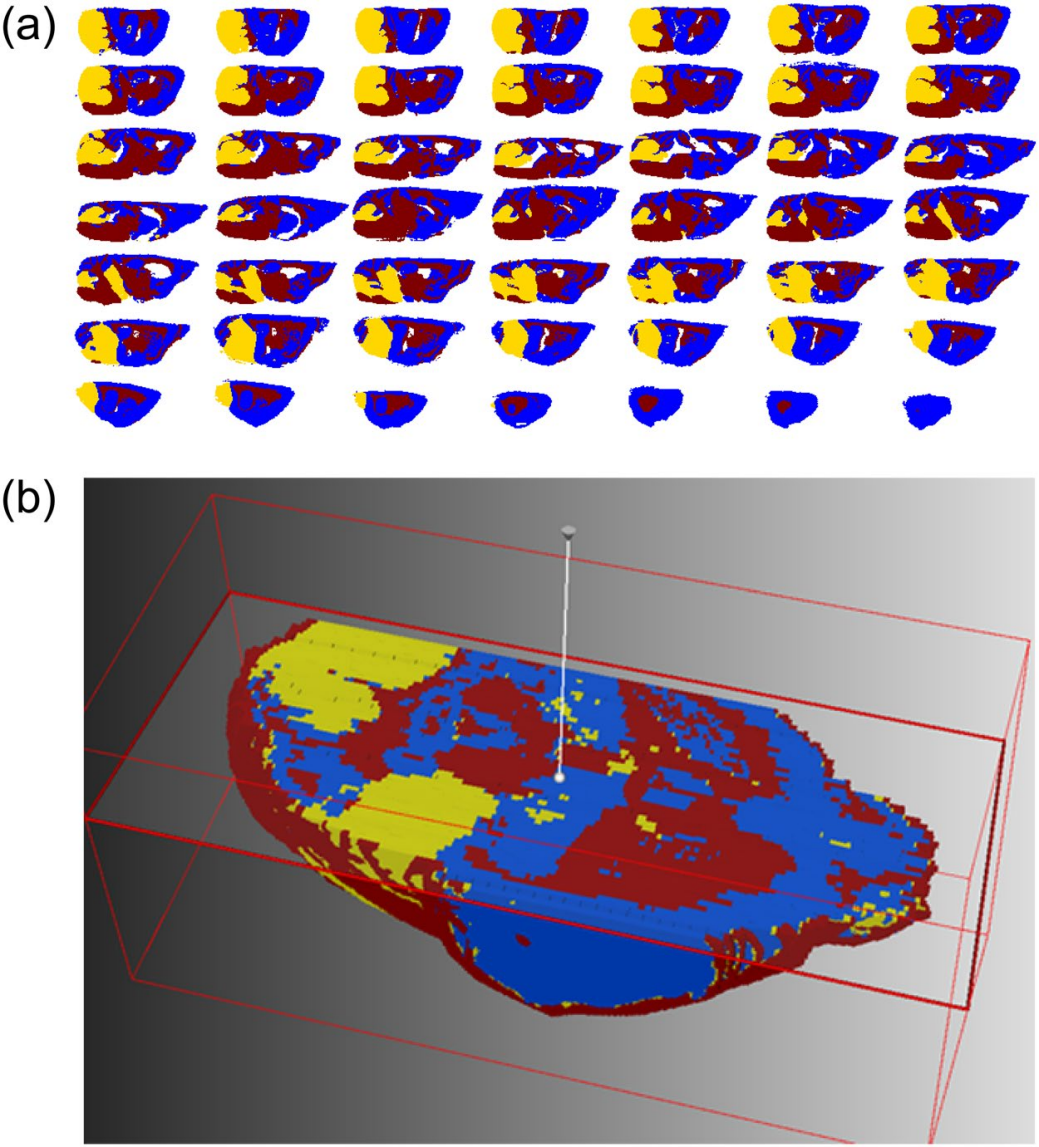
(**a**) The bisecting *k*-means segmentation of 49 sagittal tissue sections from a ND2:SmoA1 transgenic mouse brain containing a non-metastasizing cerebellum tumor showing clear delineation of grey matter (blue), white matter (red), and the primary tumor (yellow). (**b**) Visualization of the *k*-means segmentation map in a reconstructed 3D volume as a transverse virtual section.

To effectively handle such enormous datasets, we employed the SCiLS lab software application specifically designed for large and complex 3D-MALDI-ToF-MSI data^14^. The SCiLS Lab software platform is capable of aligning serial tissue sections, applying spectra-based preprocessing, image denoising^15^, statistical clustering^16^, and visualization of the data in 2- and 3-dimensions. To interpret the large dataset, we developed a supervised data mining pipeline involving semi-automated spatial segmentation of the MSI data as a means of reducing complexity. Spatial segmentation was achieved by using a clustering algorithm defining spectra as belonging to a distinct spatial group. Within the pipeline, a peak-picking algorithm was first applied to reduce the number of data points and help removing signals associated with baseline noise that negatively impacted clustering accuracy. Next, image denoising was applied to further suppress unwanted hot-spot pixels and improve the segmentation map by delineating regions based on a higher percentage of sample-derived signal^17^. Following this step, a semi-supervised bisecting *k*-means clustering algorithm that divided the MSI dataset into clusters and sub-clusters on each iteration was applied. Bisecting *k*-means clustering is better suited over conventional hierarchical clustering due to its computational efficiency and reduced memory needs, attributes that are critical for large 3D-MSI datasets^17^. The result of the segmentation analysis is a binary hierarchical tree containing nested sub regions, each defined by a unique mass spectrum. From the spatial distribution of ions at *m/z* 885.5 (Fig. 1b; red region) the 3D tumor volume was identified and validated with multiple H&E stains throughout the volume of the tissue; two of which are shown in Fig. 1c. By direct comparison with the images shown in Fig. 1 we identified the *k*-means cluster most closely representing the MB primary tumor (Fig. 2; yellow region) as opposed to the healthy grey matter (Fig. 2; blue region) and white matter (Fig. 2; red region). Supervised k-means segmentation analysis results were further validated by independent examination of tissue sections by a trained histopathologist, confirming an excellent match (SI Fig. 2).

**Figure 3:**
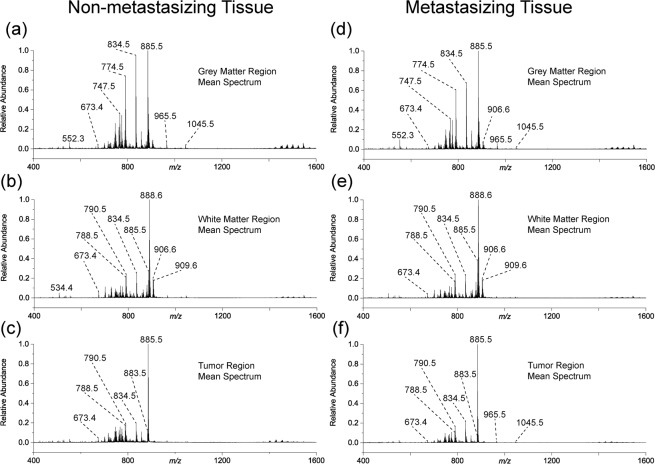
The calculated mean spectra for (**a**) grey matter, (**b**) white matter, and (**c**) tumor regions mouse brain containing a non-metastasizing medulloblastoma primary tumor and the (**d**) grey matter, (**e**) white matter, and (**f**) tumor regions in a mouse brain containing a metastasizing medulloblastoma primary tumor as defined by *k*-means segmentation. The lipidome of the tumor is comparatively very different to the grey and white matter regions based on changes in the measured relative abundances of metabolites throughout the spectrum. Differences between metastasizing and non-metastasizing tumors are subtler.

The workflow described above was then applied to six ND2:SmoA1 transgenic mice brains collected under identical conditions; three brains identified as containing a non-metastasizing primary tumor, and three brains containing a metastasizing primary tumor. Following segmentation of the mice brains into three major regions; grey matter, white matter, and tumor region, an average mass spectrum representative of each region was created. Figure 3 shows the mean mass spectra for the three segmented regions from both non-metastasizing and metastasizing MB mouse brains. The contrasting tumor lipidome is what differentiates the tumor region from the remainder of the healthy brain tissue, as evidenced by comparing the mean spectra from the three regions of one brain, e.g., Fig. 3a–c. However, comparing similar regions between different brains, i.e., comparing both the tumor region mean spectra (Fig. 3c,f) does not immediately reveal observable differences. Therefore, once the cluster-defined tumor regions from both the non-metastasizing and metastasizing primary MB tumors were identified, the associated mass spectra for all tumor clusters were statistically compared, providing a robust approach for investigating the underlying metabolic phenotype associated with MB metastasis. A total of 223 sections (3.3 TB of data) from six SmoA1-GFP mouse brains were analyzed: 105 sections from brains containing a non-metastasizing primary tumor and 118 from brains containing a metastasizing primary tumor. The number of slices varied between brains due to slight tissue shape deformations during the freezing process. For each section, a mass spectrum was recorded at every 50 µm × 50 µm pixel position, totaling 10.2 million individual spectra for the entire dataset. The average data acquisition time for each section was approximately 24 min.

To compare lipid abundance data within 441,152 individual mass spectra from non-metastasizing and 1,010,805 spectra from metastasizing primary tumor regions, we first used a ROC curve approach to evaluate each *m/z* value based on its discriminating power^18^. Area under the curve (AUC) values between 0 and 1 were obtained for each *m/z* value describing a signal’s discriminatory power based on its normalized relative abundance. In the binary comparison between non-metastasizing and metastasizing primary tumors, signals having AUC ≥ 0.6 were deemed significantly decreased in the metastasizing tumor while signals having an AUC value ≤ 0.4 were significantly more abundant. Using these AUC thresholds, ten lipids were identified as having significant differences in TIC normalized relative abundances between the two groups (Table 1). Assignment of chemical structures to the discriminant markers was accomplished using accurate mass measurements (SI Fig. 3) and MS/MS experiments (SI Fig. 4), with fragmentation patterns being compared to literature^19,20^ and the ALEX^123^ Lipid Calculator database (http://alex123.info/ALEX123/MS.php) with the fragmentation reported using their proposed nomenclature for lipid fragment ions^21^. Identified lipid species are reported using the shorthand nomenclature system outlined by Liebisch *et al*.^22^. Further univariate statistical analysis for the abundances of lipids is provided in SI Fig. 5.

**Table 1:** Differential metabolites identified using negative-ion mode 3D-MALDI-MSI with statistically-different abundances in metastasizing primary tumors compared to non-metastasizing primary tumors in SmoA1-GFP murine medulloblastoma based on individual AUC values.

Measured *m/z*	Theo. *m/z*	Mass accuracy (ppm)	AUC value*	Assigned Metabolite^†^
**Decreased in metastasizing tissue**				
673.4811	673.4814	0.4	0.65	PA (16:0_18:1)
723.4968	723.4968	0.05	0.69	PA (18:0_20:4)
726.5440	726.5443	0.4	0.61	PE (*P*-18:1/18:1)
**PE(** ***O*** **-18:2/18:1)**				
738.5078	738.5079	0.1	0.64	PE (16:0_20:4)
742.5390	742.5392	0.3	0.66	PE (18:1/18:1)
760.5131	760.5134	0.4	0.67	PS (16:0_18:1)
**Increased in metastasizing tissue**				
834.5284	834.5291	0.8	0.40	PS (18:0_22:6)
885.5489	885.5499	1.1	0.31	PI (18:0_20:4)
965.5155	965.5156	0.1	0.37	PIP (18:0_20:4)
1045.4812	1045.4820	0.8	0.36	PIP_2_ (18:0_20:4)

See SI Fig. 5 for additional statistical testing.

^*^Values ≥ 0.6 were suspected to be decreased in the metastasizing tumor and lower than ≤ 0.4, increased in metastasizing tumors.

^†^All metabolites in table were detected as their [M-H]^−^ ionic species.

Of the ten lipid species identified to be altered between non-metastasizing and metastasizing SHH MB primary tumors using ROC analysis, six were decreased and four were increased in metastasizing tissue (Table 1). The six decreased species included two phosphatidic acids (PA), three phosphatidylethanolamines (PE), and a phosphatidylserine (PS). The two PA species identified by ROC analysis [PA(16:0_18:1), PA(18:0_20:4) are not only decreased in the metastasizing tumor relative to the non-metastasizing tumor, but are also decreased in both tumors relative to neighboring healthy grey matter tissue, which shares the most similar lipidome to the MB tumor tissue. It is important to note that the same species can also be formed by in-source fragmentation during the MALDI process, and would be indistinguishable from the endogenous component. Many glycerophospholipids have the potential to form a PA fragment, with different glycerophospholipids able to contribute to a single PA mass channel if they share identical fatty acyl chain compositions. However, in-source fragmentation contributions are expected to be similar across metastasizing and non-metastasizing tissue slices, but it is still possible that the PA abundances found in this study contain contributions from both endogenous and in-source fragmentation.

Several studies have identified PAs as being important signaling molecules in cell migration and metastasis due to their growth factor–like properties and their role in tumor cell chemotaxis^23^. Overexpression of the membrane protein phospholipase D (PLD) that converts phosphatidylcholines to PAs by cleaving the choline group can result in increased PA levels. Elevated total PLD activity has been observed in all cancers where it has been examined, such as gastric, colorectal, kidney, stomach, esophagus, lung, and breast^23–25^. Our observations of decreased PAs are seemingly contrary to these findings. However, it has been shown that temporal changes in PA levels critically regulate the activity of ERK, mTOR and the Ras superfamily of small GTPases by modulating membrane localization and activity of small GTPase regulatory proteins^26–28^. It is thus possible that relative alterations in the levels of PA, rather than over- or under expression in general, more precisely regulate cell signaling important to cancer cell proliferation, cell migration and tumor metastasis. It is also possible that PLD may be overexpressed in MB, however there may be elevated biochemical processes downstream involving the metabolism/degradation of PAs causing a net reduction of this class of metabolite. Alternatively, PLD activity might be suppressed in MB, resulting in our observed decreased PA abundances. Regardless, the two PAs identified in this study have relative distributions unlike many of the other lipids detected. Many lipids are generally present in elevated levels in the cancer tissue due to their increased production by cancer cells to satisfy their fundamental needs to expand and disseminate. The net relative reduction of these PA species, particularly in the metastasizing tumor compared to non-metastasizing, suggests these two species may be an important intermediate in lipid metabolism associated with brain cancer metastasis.

Of the six lipids identified as being decreased in the metastasizing tumor relative to the non-metastasizing tumor, four contain an 18:1 fatty acyl chain as either one or both of the chains linked to the lipid backbone. This is the largest commonality between the lipids observed to be decreased in the metastasizing tumors, identified across an array of lipid classes including; PA(16:0_18:1), PE(18:1/18:1), PS(16:0_18:1), PE(P-18:1/18:1) and/or PE(O-18:2/18:1). The role these lipids play in the process of tumor metastasis is unclear as the biological function of most lipids at this level of chemical specificity is poorly understood. However, the decreased abundance of these lipids in the metastasizing tumors may be the indirect result of changes in metabolic pathways involving the incorporation/release of oleic acid as a free fatty acid. Not surprisingly, there have been conflicting reports on the influence oleic acid has on cancer progression and metastasis, as the study of lipids such as this, which are involved in numerous and varied cellular processes, is extremely difficult. For example, the same study found that oleic acid fed highly metastatic carcinoma cells and boosted malignancy, whereas it actually inhibited cell growth and survival in low metastatic carcinoma cells^29^. Oleic acid has also been reported to promote migration of breast cancer cells^30^ while also being linked to cell apoptosis and autophagy in tongue squamous cell carcinomas^31^.

Among the lipids observed to be increased in metastasizing primary tumors are the phosphatidylinositol PI(18:0_20:4) and two phosphoinositides PIP(18:0_20:4) and PIP_2_(18:0_20:4) (Table 1). These three lipids are all structurally related, containing the same fatty acyl chain composition and core head group, and differing only by the degree of phosphorylation on the inositol moiety. PIPs are generated by phosphoinositide kinases (PIKs) to convey signals from the cell surface to the cytoplasm, and are downstream effectors on multiple pathways involved in the survival and growth of normal cells^32,33^. Dysregulation of the PI3K signaling pathway, most commonly caused by alteration of the *PTEN* gene, has been widely associated with oncogenesis in humans due to unbridled cell growth and proliferation^33,34^. All three lipids – participants in the same signaling pathway – were observed to be elevated in metastasizing tumors relative to non-metastasizing tumors, therefore providing strong evidence that this pathway is upregulated in medulloblastoma metastasizing tissue.

**Figure 4:**
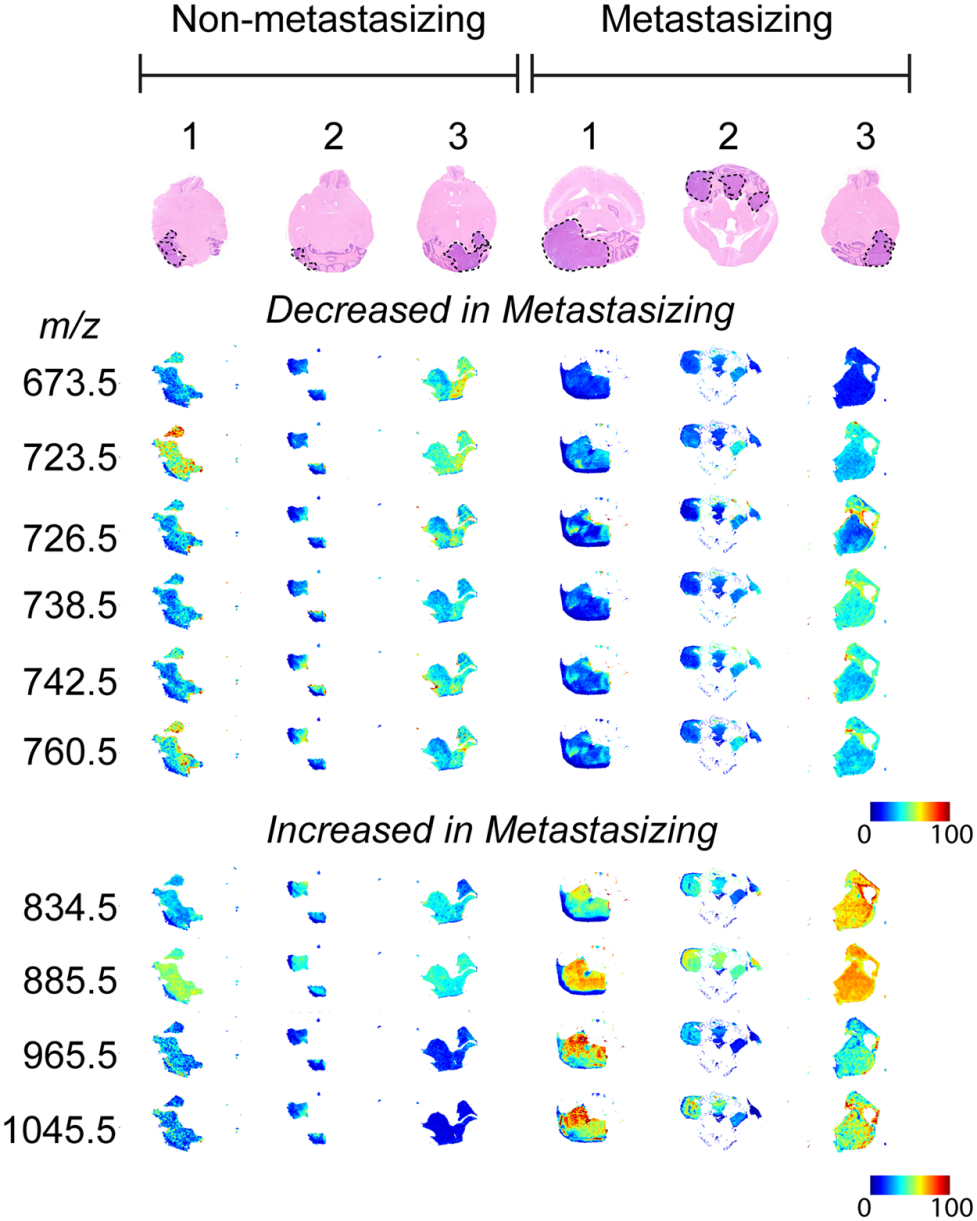
Optical images of a single transverse tissue section following H&E staining (tumor regions defined by black dotted line) for each of the six mouse brains used in the comparative study and the corresponding extracted-ion images for the ten lipids identified in Table 1 shown as 2-dimensional false-color plots. For each *m/z* values visualized distribution, TIC normalization was applied across all six tumor regions for direct comparison. The extracted-ion images highlight the heterogeneous spatial distribution within each tumor region and also general comparative changes across the six individual tumors.

Extracted-ion images for the ten identified lipids within the tumor region are shown in Fig. 4 for a single section chosen at random from each brain. For direct comparison, false-color images for a single mass channel were TIC normalized across the 6 brains. Automatic hotspot removal was also applied using a low quantile threshold of 0% and a high quantile threshold of 99%. The hotspot removal function automatically adjusts the color scale to better visualize changes in abundance for the bottom 99% of pixels. For each lipid identified, a heterogeneous spatial distribution in 3-dimensions was observed, particularly for those detected at low abundances. This observation illustrates the crucial importance of 3D-MSI for accurate identification of biologically relevant lipids. Whereas 2D-MSI visualized primary MB tumor tissue present in almost every section analyzed, 3D-MSI images detailed the heterogeneity of the relevant lipids throughout the tumor volume. If conventional 2D-MSI had been employed, single or multiple tissue sections may have been selected that may not be truly representative of the heterogeneous tumor lipid profiles, possibly resulting in discriminant lipids going undetected.

## Methods

### Materials

Methanol (LC-MS grade), chloroform (HPLC grade), Ethanol (HPLC grade), norharmane (crystalline), and red phosphorus (≥99.99% purity) were purchased from Sigma Aldrich (Zwijndrecht, The Netherlands) and used without further purification.

### Generation of SmoA1-GFP mice specimens

ND2:SmoA1 transgenic mice were purchased from The Jackson Laboratories (Bar Harbor, ME, USA). To generate ND2:SmoA1-GFP mice for this study, homozygous ND2:SmoA1 mice (SmoA1) were crossed with Math1-GFP reporter mice kindly provided by Dr. Tracy-Ann Read, Emory Winship Cancer Institute. The F1 (SmoA1–Math-GFP heterozygous) mice were back-crossed with SmoA1 mice. Real-time PCR was used to select the F2 mice homozygous for SmoA1 as well the Math-GFP reporter gene. The selected F2 mice then were back-crossed with SmoA1 for three more generations to yield the final SmoA1-GFP mice. Using fluorescence microscopy, the primary MB tumor of the cerebellum and the metastases to the distal brain and spinal cord are readily visualized by positive detection of GFP (SI Fig. 1). Metastasis occurs in approximately one-third of mice. This has been confirmed by our previous experience in MR imaging well over 100 mice between 4 to 48 weeks of life. Micrometastasis by GFP detection on dissection of the brain and spine can be observed as early as 8 weeks of life, and in mice with metastasis detected by MR imaging, all have been found to have concomitant GFP-positive micrometastasis, while none of the mice without evidence of metastasis by MR imaging between 24–48 weeks of life had evidence of GFP-positive micrometastasis. This indicates that the absence of GFP-positive micrometastasis beyond 24 weeks of life is a highly robust marker for the non-metastasizing tumor phenotype and suggests that the SmoA1-GFP mouse is a reliable model for the investigation of these two distinct phenotypes. To ensure that the primary tumors we evaluated for metabolomic signatures were phenotypically metastasizing vs. non-metastasizing and did not harbor occult micrometastasis that would subsequently develop to overt metastasis, mice were sacrificed and evaluated at 36–38 weeks of life, well beyond the time point that GFP-positive micrometastasis, if present, would be expected to be readily detected. All animals were maintained in the animal facility at Emory University and used in accordance with protocols approved by the Emory Institutional Animal Care and Use Committee.

### Tissue preparation

For tissue analysis, whole brains of SmoA1-GFP mice were dissected immediately after the animals were sacrificed, and their brains frozen by placing the tissue in an aluminum foil boat on top of an acetone/dry ice bath (−78 °C). The frozen tissues were stored at −80 °C until ready for cryosectioning to avoid metabolite degradation. The spinal cord was isolated and carefully inspected by fluorescence microscopy for the identification of metastasizing tumors (GFP positive) immediately after mice were sacrificed. For 3D-MSI analyses, three whole brains containing a primary tumor with extensive spinal metastases were obtained from SmoA1-GFP mice, and three whole brains containing a primary tumor without detectable metastases were obtained from SmoA1-GFP mice. Prior to sectioning the tissue, the samples were placed within the cryotome for 20 min to equilibrate to the cutting temperature (from −80 to −20 °C). Entire mouse brains were sliced into 10 µm-thick sections with sections kept at every 150 µm depth through the tissue. From the six SmoA1-GFP mice brains selected, we retained a total of 223 sections for 3D reconstructions. The retained sections were thaw-mounted onto indium tin oxide (ITO)-coated glass slides and stored at −80 °C until analyzed.

### MALDI-MS sample preparation

For large sample batches (30–50 mouse tissue sections per brain), the sample preparation method was streamlined to reduce the sample preparation time and inter-sample variability as much as possible. This was achieved by mounting as many tissue sections as possible on each glass slide (typically 4 or 5 sections per slide) and applying matrix to 5 slides as one batch. Firstly, the ITO slides were dried under a flow of nitrogen for 10 s and then coated with norharmane (7 mg/mL in 70% CHCl_3_, 30% MeOH) flowing at 0.12 mL/min using a TM-Sprayer (HTX, Chapel Hill, NC, USA). The matrix solution was spray-coated onto the tissue sections using a pneumatically-assisted spray heated at 30 °C that robotically moves in a predefined crisscross pattern traversing the sample with orthogonal passes. Each sample was coated with four layers of matrix that included 30 s of drying time between layers. The robotic sprayer provided highly reproducible and uniform deposition of matrix across multiple slides, a crucial step for the integration of (or comparison between) multiple MS images. Using this method, 5 glass slides (25 × 75 mm) containing a total of 20–25 brain sections are able to be coated within 15 mins. This equates to approximately 45 s per brain section. After matrix was applied, small registration marks were made on the slide with a diamond-tip pen, 1 µL of red phosphorus calibration solution (100% EtOH; saturated) pipetted onto the slide near the tissue, and an optical image acquired using a Nikon Super COOLSCAN 5000 ED film scanner (Nikon, Melville, NY, USA).

### Mass spectrometry analysis

Tissue sections were analyzed in negative-ion mode using a Bruker rapifleX MALDI Tissuetyper ToF mass spectrometer operating in reflectron mode and equipped with a frequency tripled 355 nm Nd:YAG laser at repetition rates up to 10 kHz. Laser power and laser focus position were manually fine-tuned before each acquisition to ensure optimal data quality and comparable signal intensity. This tuning was performed on an area containing only matrix on the glass slide and tuning the parameters so the signal attributable to matrix ionic species were within ± 10% of the peak intensity compared to the previous acquisition. Instrument parameters were optimized for maximum acquisition speed while maintaining a resolving power above 15,000 at m/z 885. Imaging experiments were controlled by the flexImaging 4.0 software (Bruker Daltonics, Billerica, MA, USA) with a laser raster size of 40 μm^2^ and a stage motion (i.e., pixel size) of 50 μm^2^. At each raster position, 200 laser shots were summed to generate a representative spectrum for each pixel, with the digitizer sampling rate at 1.25 GS/s. Spectra were acquired in the *m/z* 400–1600 range at each pixel position. The average data acquisition time per tissue section was 24 min with acquisition speeds of ~50 pixels/s. To minimize mass drift and peaks shifting between acquisitions, the instrument was externally calibrated before each imaging experiment using red phosphorus as it provides singly charged, monoisotopic cluster ions spanning the 200–3000 Da mass range in both positive- and negative-ion modes^35^. Accurate mass and MS/MS measurements for lipid identification purposes were performed in negative-ion mode on an Orbitrap Elite mass spectrometer (Thermo Fisher Scientific GmbH, Bremen, Germany) operating at the resolving power setting 240,000 at m/z 400 and coupled to an intermediate pressure MALDI source based on a dual ion funnel design (Spectroglyph LLC, Kennewick, WA, USA). Tandem MS spectra were acquired from the tumor region by selecting ions of interest within a mass window of 0.7 Da and performing higher-energy collision induced dissociation (HCD) with a normalized collision energy of 55 arbitrary units and an activation time of 10 ms, detecting the product ions in the orbitrap with 240,000 resolving power at m/z 400. Tandem MS spectra were averaged from a minimum of 250 scans. To ensure confident lipid molecular assignments, product ions corresponding to the neutral loss of the fatty acyl chains accurate to < 2 ppm were detected for all but the ion at m/z 1045.5 due to the low ion abundance.

### Data processing, spatial segmentation, and discriminant analysis

All data processing was performed using the software SCiLS Lab (SCiLS GmbH, Bremen, Germany) on a computer system with an Intel Core i7 processor running Microsoft Windows 7 64-Bit operating system and 64 GB of RAM. Mass spectra were preprocessed during import into SCiLS Lab using baseline removal by iterative convolution(1) and Total Ion Count (TIC) spectral normalization (2). These processing methods are common practice in MSI workflows and are well-established in the MSI community (2). Three-dimensional registration of serial sections was performed based on the ion image of m/z 790.5 after import of the data. For 3D visualization, a section thickness of 150 µm was used. For subsequent data analysis, a minimum interval width of 500 mDa around the average peak center was used to account for peak shifts throughout the experiment. Hypothesis testing was performed with Metaboanalyst 3.0 (http://www.metaboanalyst.ca/) using the non-parametric Mann–Whitney–Wilcoxon test. Semi-automatic 3D spatial segmentation by means of image denoising and subsequent statistical clustering was performed to generate an overview of the dataset. This analysis allowed to quickly delineate regions corresponding to the white matter anatomical region, the grey matter anatomical region, and the primary tumor region^17^. Based on the regions delineating metastasizing and non-metastasizing primary tumors, we used receiver operating characteristic (ROC) curves to find discriminating *m/z* markers. ROC curves assess the discrimination quality of specific *m/z* values for populations defined through two groups. It is calculated based on the statistical specificity and sensitivity when the abundance of a single *m/z* value represents the discrimination rule^18^.

### H&E staining

Following MALDI-MSI analysis, select tissue sections were stained with hematoxylin and eosin to facilitate histological discrimination between healthy brain tissue and tumor tissue regions. Norharmane matrix was removed from the sections with a 5 min wash in 70% ethanol, followed by brief rinsing in distilled water. Slides were then stained in Harris hematoxylin solution for 8 mins followed by rinsing with running tap water for 5 min. Sections were then counterstained in eosin-phloxine solution for 1 min, followed by conventional dehydration and coverslipping, i.e., dehydration through 70% and 96% ethanol (2 changes of 5 min each), clearing steps in 2 changes of xylene (5 min each), and mounting with xylene based mounting medium. Optical images of stained tissues were acquired using a Nikon Super COOLSCAN 5000 ED film scanner (Nikon, Melville, NY, USA) at a 4000 dpi true optical resolution.

## Conclusions

To our knowledge, this is the first report of the use of mass spectrometry to decipher specific lipid alterations associated with the development of SHH MB and MB metastasis. By employing a rapid 3D-MSI approach, we have, for the first time, identified ten lipids that are differentially expressed within non-metastasizing and metastasizing SHH MB primary tumors. Due to the low abundance and heterogeneity of these lipids within the tissue, identifying these lipids was only successful by imaging the full 3D volume of the brain and specifically segmenting the tumor regions. The ten lipids identified represent prime candidates for validation in human MB tissues and for further investigation with respect to the potential functional mechanism of these lipids in the promotion of metastasis in human and murine SHH MB. Whether these lipids are specific to SHH MB metastasis, or MB metastasis in general, will also need to be verified in future studies. Prior studies seeking to understand the regulation of MB metastasis have typically introduced targeted or transposon-mediated random genetic integration to investigate the impact of these genetic alterations on metastasis. However, it is unclear whether these additional genetic modifications alter the natural progression of MB and accurately reflect the human disease. This study offers the advantage of employing an unbiased global interrogation, and a genetically unmodified model of the disease, other than the necessary initiating oncogenic mutation and a GFP reporter to identify metastases, to uncover candidate metabolites in the promotion of metastasis. This study also does not employ chemical agents or genetic manipulation that may suppress tumor growth, and in turn reduce metastasis, rather than address the associations and mechanisms of metastasis directly.

## Supplementary information

Supplementary Information

## Data Availability

The datasets generated during and/or analyzed during the current study are available from the corresponding author on reasonable request.
