# Supplementary material for: Three-Dimensional Mass Spectrometry Imaging Identifies Lipid Markers of Medulloblastoma Metastasis
Source: Sci Rep. 2019 Feb 18;9:2205. doi: 10.1038/s41598-018-38257-0 (PMC6379434; doi:10.1038/s41598-018-38257-0)
Supplement: Supplementary file 1 — Supplementary Information [file 41598_2018_38257_MOESM1_ESM.pdf]

Three-Dimensional Mass Spectrometry Imaging Identifies Lipid Markers of Medulloblastoma Metastasis

Martin R. L. Paine[1,2], Jingbo Liu[3], Danning Huang[1], Shane R. Ellis[2], Dennis Trede[4], Jan H. Kobarg[4], Ron M.A. Heeren[2*], Facundo M. Fernández[1,5,6*], Tobey J. MacDonald[3*]

[1]School of Chemistry and Biochemistry, Georgia Institute of Technology, Atlanta, GA 30332, USA.

[2]Maastricht Multimodal Molecular Imaging Institute, Division of Imaging Mass Spectrometry, Maastricht University, Maastricht 6229ER, The Netherlands.

[3]Aflac Cancer and Blood Disorders Center, Department of Pediatrics, Emory University School of Medicine, Atlanta, GA 30322, USA.

[4]SCiLS Lab, Bremen 28359, Germany.

[5]Integrated Cancer Research Center, Georgia Institute of Technology, Atlanta GA 30332 USA.

[6]Institute of Bioengineering and Biosciences, Georgia Institute of Technology, Atlanta, GA 30332, USA.

*Co-corresponding authors. Please address all correspondence to:
tobey.macdonald@emory.edu, facundo.fernandez@chemistry.gatech.edu,
r.heeren@maastrichtuniversity.nl

Supplementary Information

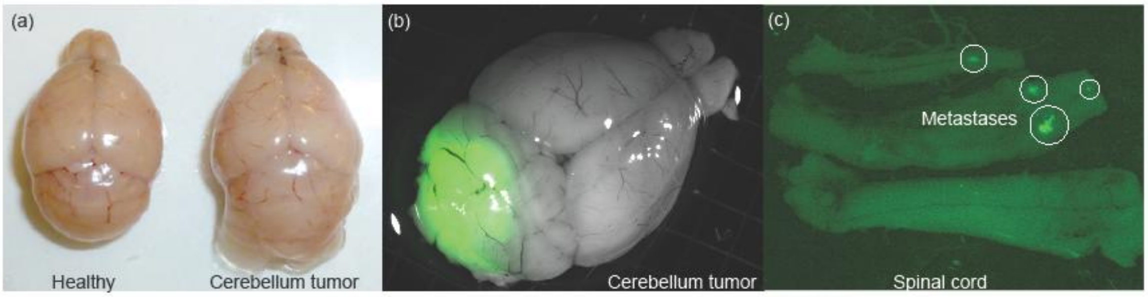

Supplementary Figure S1. (a) Optical image comparing a healthy mouse brain (left) and a ND2:SmoA1 transgenic mouse brain containing a cerebellum tumor (right). (b) The primary medulloblastoma tumor in the cerebellum easily visualized by positive detection of green fluorescent protein (GFP) using fluorescence microscopy. (c) Positive identification of a metastasizing primary tumor in the cerebellum by visualizing the metastases in the spinal cord with the detection of GFP (white circles) using fluorescence microscopy.

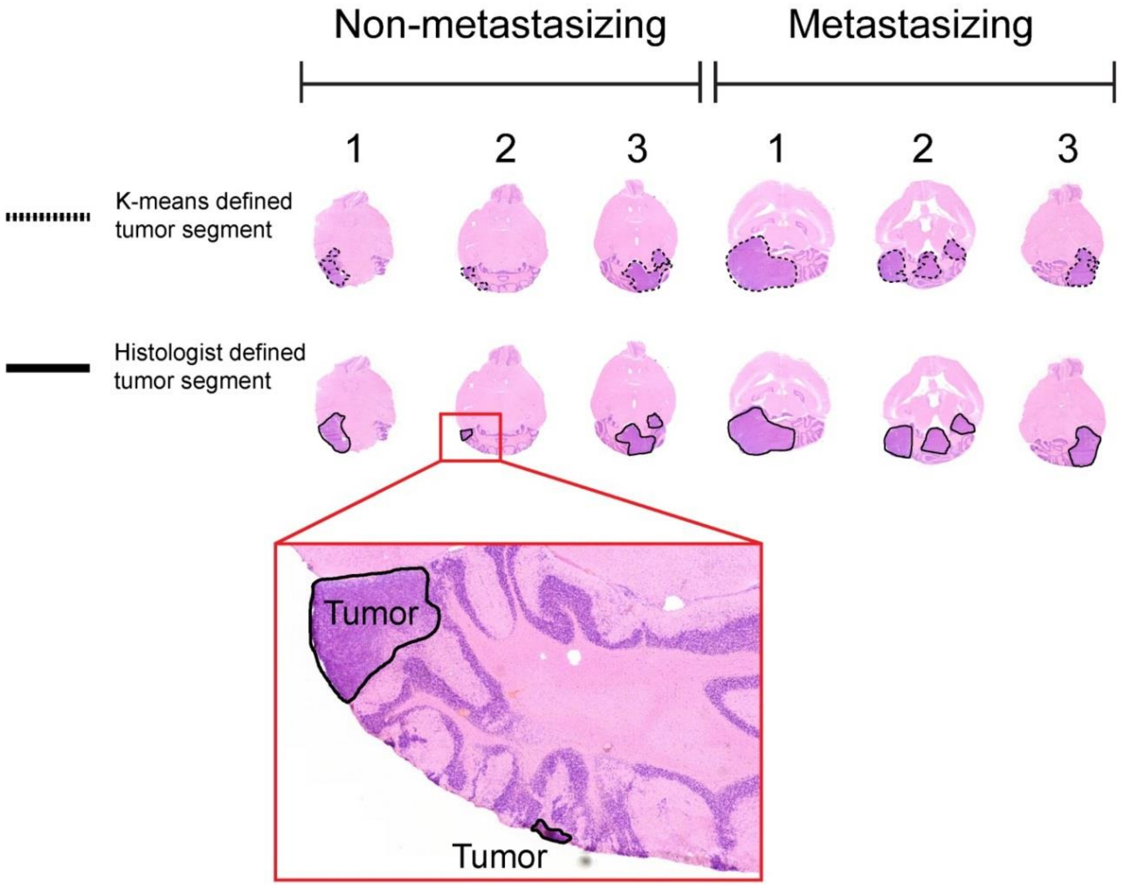

Supplementary Figure S2. Validation of the semi-supervised bisecting k-means tumor segmentation by comparison with histological annotation of the H&E stained tumor tissue. K-means defined tumor segments align perfectly with the histology defined tumor segments, as determined by independent expert neuropathology review of the tissue using light microscopy. Inset – magnified area of non-metastasizing brain sample no. 2 showing the major tumor region identified by the expert neuropathologist on first inspection of the tissue (top left), and the minor tumor segment (bottom right) not initially identified. This minor segment was identified by the k-means segmentation method, and was subsequently confirmed by the neuropathologist after being directed to the specific area of tissue in question and performing a second closer inspection under higher magnification, thus exemplifying the sensitivity and usefulness of the k-means segmentation method.

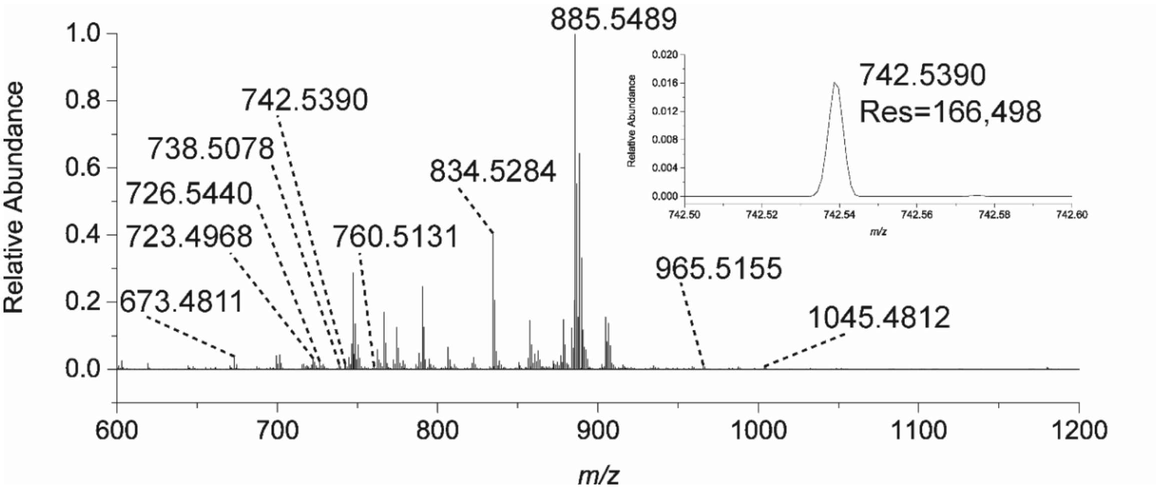

Supplementary Figure S3. Negative-ion mode MALDI-FTMS spectrum acquired from a non-metastasizing ND2:SmoA1mouse brain tumor tissue using an orbitrap Elite mass spectrometer. The spectrum was acquired with a mass resolving power setting of 240,000 at m/z 400. Inset – an expanded view of the mass range between m/z 742.50 and 742.60.

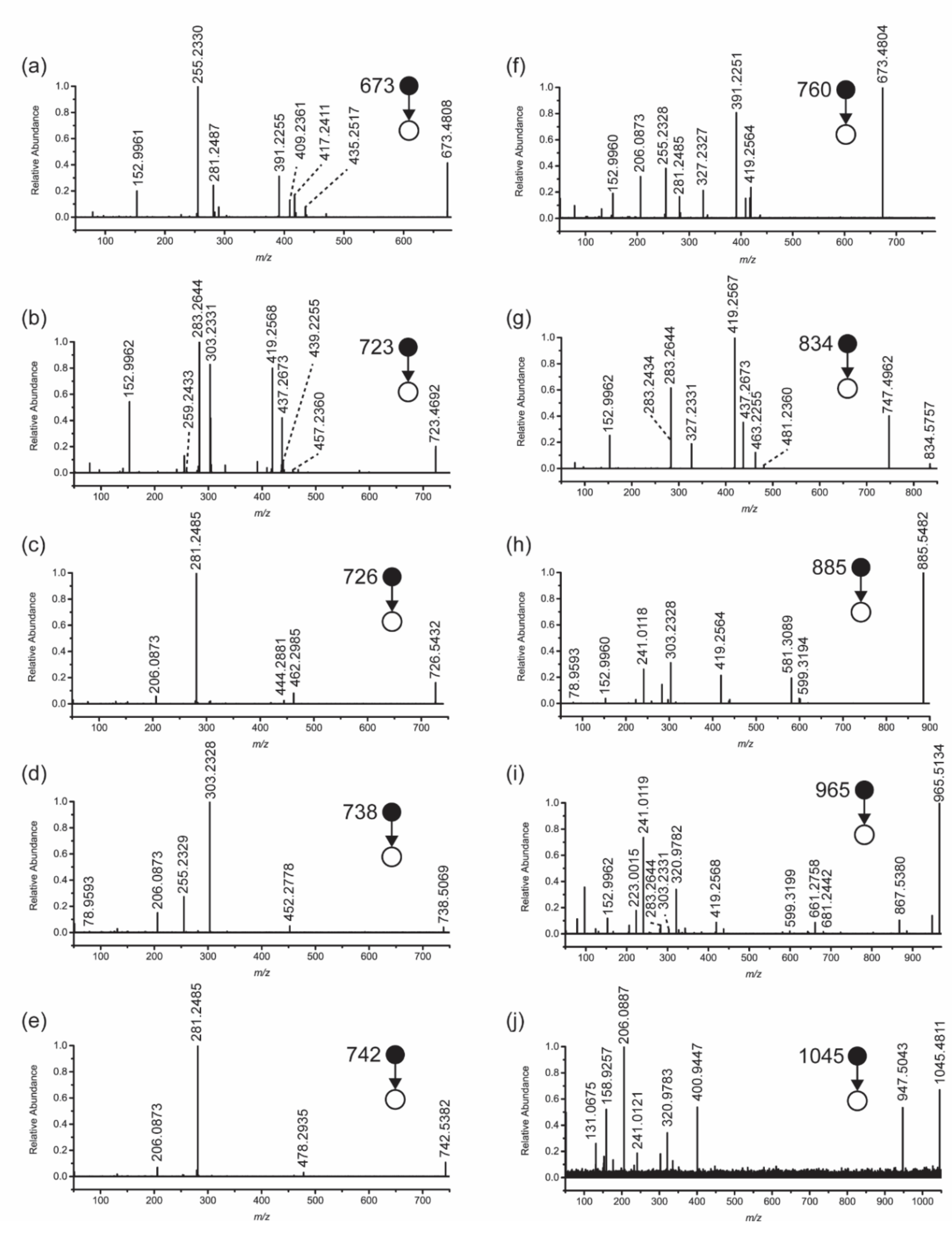

Supplementary Figure S4. Higher energy collision induced dissociation (HCD) tandem MS spectra acquired using negative-ion mode MALDI-FTMS on an Orbitrap Elite mass spectrometer for each lipid listed in Table 1. The spectra shown are the average of over 250 individual scans and were used to confirm metabolite identification in conjunction with accurate mass measurements.

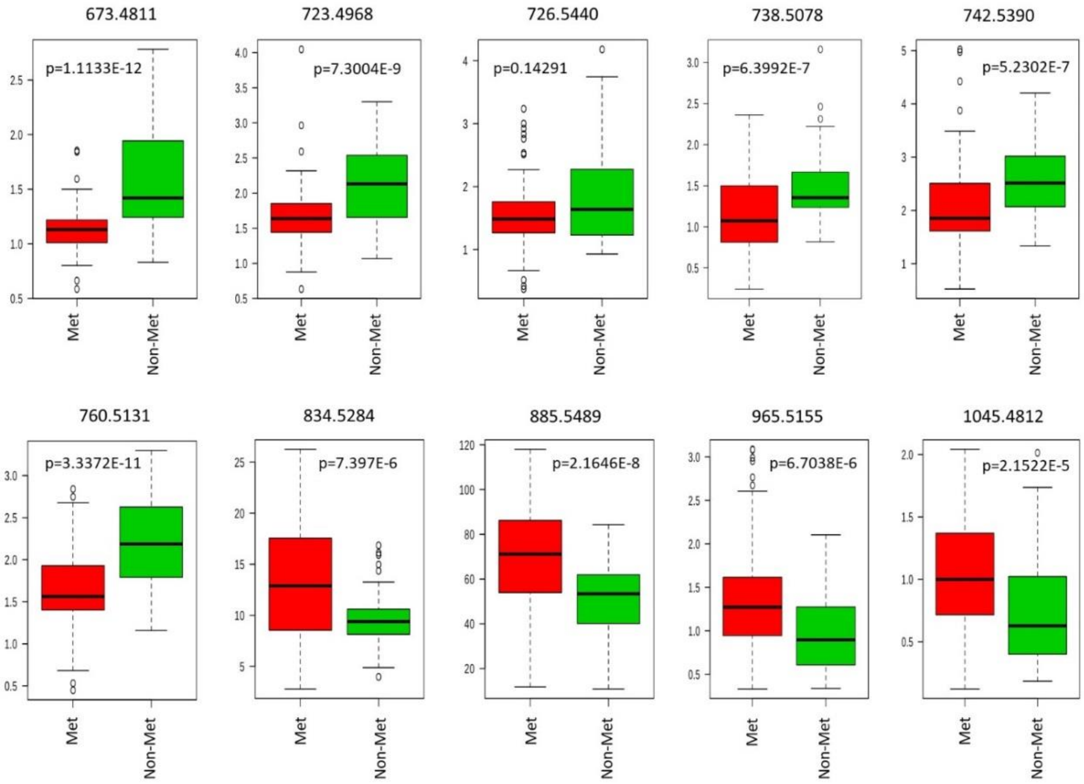

Supplementary Figure S5. Box plots and corresponding p-values obtained through hypothesis testing for the lipid markers in Table 1. A Mann–Whitney–Wilcoxon test as provided in Metaboanalyst 3.0 was used. Normalized lipid abundances in all non-metastasizing 2D tissue sections containing tumor tissue were compared against abundances in tissue sections containing metastasizing tumor. A total of 9 out of 10 lipid species showed statistically significant differences, with the exception of m/z 726.5440 (PE(P-18:1/18:1) or PE(O-18:2/18:1), which was marginal (p=0.14291).

Supplementary Table S1. Assignment of MS/MS product ions shown in SI Figure 4 and their relative error (ppm) based on the ALEX123 Lipid Calculator database (http://alex123.info/ALEX123/MS.php) with the fragmentation products reported using their proposed nomenclature for lipid fragment ions.(1)

**Precursor *m/z***	**Theoretical *m/z***	**error (ppm)**	**Product ion *m/z***	**Fragmentation**	**Fragment-ion molecular formula**	**Theoretical Fragment *m/z***	**error (ppm)**
673.4808	673.4814	0.9	435.2517	-FA 16:0(-H)	C21H40O7P	435.251714	0.0
673.4808	673.4814	0.9	417.2411	-FA 16:0(+HO)	C21H38O6P	417.24115	0.1
673.4808	673.4814	0.9	409.2361	-FA 18:1(-H)	C19H38O7P	409.236064	0.1
673.4808	673.4814	0.9	391.2255	-FA 18:1(+HO)	C19H36O6P	391.2255	0.0
673.4808	673.4814	0.9	281.2487	FA 18:1(+O)	C18H33O2	281.248604	0.3
673.4808	673.4814	0.9	255.233	FA 16:0(+O)	C16H31O2	255.232954	0.2
673.4808	673.4814	0.9	152.9961	Glycerol-3-phosphate -H2O	C3H6O5P	152.995834	1.7
723.4962	723.4970	1.1	457.236	-FA 18:0(-H)	C23H38O7P	457.236064	0.1
723.4962	723.4970	1.1	439.2255	-FA 18:0(+HO)	C23H36O6P	439.2255	0.0
723.4962	723.4970	1.1	437.2673	-FA 20:4(-H)	C21H42O7P	437.267364	0.1
723.4962	723.4970	1.1	419.2568	-FA 20:4(+HO)	C21H40O6P	419.2568	0.0
723.4962	723.4970	1.1	303.2331	FA 20:4(+O)	C20H31O2	303.232954	0.5
723.4962	723.4970	1.1	283.2644	FA 18:0(+O)	C18H35O2	283.264254	0.5
723.4962	723.4970	1.1	259.2433	FA 20:4(-CO)	C19H31	259.243125	0.7
723.4962	723.4970	1.1	152.9962	Glycerol-3-phosphate -H2O	C3H6O5P	152.995834	2.4
726.5440	726.5443	0.4	462.2985	-FA 18:1(-H)	C23H45NO6P	462.298998	1.1
726.5440	726.5443	0.4	444.2881	-FA 18:1(+HO)	C23H43NO5P	444.288434	0.8
726.5440	726.5443	0.4	281.2485	FA 18:1(+O)	C18H33O2	281.248604	0.4
726.5440	726.5443	0.4	279.2328	FA 18:2(+O)	C18H31O2	279.232954	0.6

738.5078	738.5079	0.2	452.2778	-FA 20:4(-H)	C21H43NO7P	452.278263	1.0
738.5078	738.5079	0.2	303.2328	FA 20:4(+O)	C20H31O2	303.232954	0.5
738.5078	738.5079	0.2	259.2429	FA 20:4(-CO)	C19H31	259.243125	0.9
738.5078	738.5079	0.2	255.2329	FA 16:0(+O)	C16H31O2	255.232954	0.2
742.5390	742.5392	0.3	478.2935	-FA 18:1(-H)	C23H45NO7P	478.293913	0.9
742.5390	742.5392	0.3	460.283	-FA 18:1(+HO)	C23H43NO6P	460.283349	0.8
742.5390	742.5392	0.3	281.2485	FA 18:1(+O)	C18H33O2	281.248604	0.4
760.5131	760.5134	0.4	673.4804	-PS(87)	C37H70O8P	673.48138	1.5
760.5131	760.5134	0.4	417.2407	-FA 16:0(+HO) -PS(87)	C21H38O6P	417.241149	1.1
760.5131	760.5134	0.4	409.2356	-FA 18:1(-H) -PS(87)	C19H38O7P	409.236064	1.1
760.5131	760.5134	0.4	391.2251	-FA 18:1(+HO) -PS(87)	C19H36O6P	391.225499	1.0
760.5131	760.5134	0.4	281.2485	FA 18:1(+O)	C18H33O2	281.248604	0.4
760.5131	760.5134	0.4	255.2328	FA 16:0(+O)	C16H31O2	255.232954	0.6
760.5131	760.5134	0.4	152.996	PS(153)	C3H6O5P	152.995834	1.1
834.5284	834.5291	0.8	747.4962	-Serine	C43H72O8P	747.49703	1.1
834.5284	834.5291	0.8	481.236	-FA 18:0(-H) -Serine	C25H38O7P	481.236064	0.1
834.5284	834.5291	0.8	463.2255	-FA 18:0(+HO) -Serine	C25H36O6P	463.225499	0.0
834.5284	834.5291	0.8	437.2673	-FA 22:6(-H) -Serine	C21H42O7P	437.267364	0.1
834.5284	834.5291	0.8	419.2567	-FA 22:6(+HO) -Serine	C21H40O6P	419.256799	0.2
834.5284	834.5291	0.8	327.2331	FA 22:6(+O)	C22H31O2	327.232954	0.4
834.5284	834.5291	0.8	283.2644	FA 18:0(+O)	C18H35O2	283.264254	0.5
834.5284	834.5291	0.8	283.2434	FA 22:6(-CO)	C21H31	283.243125	1.0
834.5284	834.5291	0.8	152.9962	Glycerol-3-phosphate -H2O	C3H6O5P	152.995834	2.4
885.5489	885.5499	1.1	619.2879	-FA 18:0(-H)	C29H48O12P	619.288887	1.6

885.5489	885.5499	1.1	601.2776	-FA 18:0(+HO)	C29H46O11P	601.278323	1.2
885.5489	885.5499	1.1	599.3194	-FA 20:4(-H)	C27H52O12P	599.320187	1.3
885.5489	885.5499	1.1	581.3089	-FA 20:4(+HO)	C27H50O11P	581.309623	1.2
885.5489	885.5499	1.1	457.2355	FA 20:4(+C3H7O6P)	C23H38O7P	457.236064	1.2
885.5489	885.5499	1.1	439.225	FA 20:4(+C3H5O5P)	C23H36O6P	439.225499	1.1
885.5489	885.5499	1.1	437.2669	FA 18:0(+C3H7O6P)	C21H42O7P	437.267364	1.1
885.5489	885.5499	1.1	419.2564	FA 18:0(+C3H5O5P)	C21H40O6P	419.2568	1.0
885.5489	885.5499	1.1	303.2328	FA 20:4(+O)	C20H31O2	303.232954	0.5
885.5489	885.5499	1.1	283.2641	FA 18:0(+O)	C18H35O2	283.264254	0.5
885.5489	885.5499	1.1	259.243	FA 20:4(-CO)	C19H31	259.243125	0.5
885.5489	885.5499	1.1	259.0223	PI(259)	C6H12O9P	259.022443	0.6
885.5489	885.5499	1.1	241.0118	PI(241)	C6H10O8P	241.011878	0.3
885.5489	885.5499	1.1	223.0013	PI(223)	C6H8O7P	223.001313	0.1
885.5489	885.5499	1.1	152.996	PI(153)	C3H6O5P	152.995834	1.1
965.5155	965.5156	0.1	867.538	-H3PO4	C47H80O12P	867.5387	0.8
965.5155	965.5156	0.1	681.2442	-FA 18:0(+HO)	C29H47O14P2	681.2441	0.1
965.5155	965.5156	0.1	661.2758	-FA 20:4(+HO)	C27H51O14P2	661.2754	0.6
965.5155	965.5156	0.1	599.3199	-FA 20:4(+HO) -PO3	C21H40O6P	599.3196	0.5
965.5155	965.5156	0.1	419.2568	-FA 20:4(+HO) - Phosphoinositol(242)	C21H40O6P	419.2568	0.0
965.5155	965.5156	0.1	303.2331	FA 20:4(+O)	C20H31O2	303.2330	0.3
965.5155	965.5156	0.1	283.2644	FA 18:0(+O)	C18H35O2	283.2643	0.4
965.5155	965.5156	0.1	241.0119	Inositol phosphate ion -H2O	C6H10O8P	241.0119	0.0
965.5155	965.5156	0.1	223.0015	Inositol phosphate ion -2(H2O)	C6H8O7P	223.0013	0.9
965.5155	965.5156	0.1	152.9962	Glycerol-3-phosphate -H2O	C3H6O5P	152.9958	2.6
1045.4812	1045.4820	0.7	947.5043	-H3PO4	C47H81O15P2	947.5050	0.7
1045.4812	1045.4820	0.7	400.9447	Inositol trisphosphate -H2O	C6H12O14P3	400.9440	1.7

1045.4812	1045.4820	0.7	320.9783	Inositol bisphosphate -H2O	C6H11O11P2	320.9776	2.2
1045.4812	1045.4820	0.7	241.0121	Inositol phosphate ion -H2O	C6H10O8P	241.0119	0.8
1045.4812	1045.4820	0.7	152.9962	Glycerol-3-phosphate -H2O	C3H6O5P	152.9958	2.6

Supplementary References

1. Pauling JK, *et al.* (2017) Proposal for a common nomenclature for fragment ions in mass spectra of lipids. PLOS ONE 12(11):e0188394.
